# Low-cost male urogenital simulator for penile implant surgery training: a 3D printing approach

**DOI:** 10.1186/s41205-024-00248-5

**Published:** 2025-01-03

**Authors:** Zixi Wang, Carlo Saija, Nicholas Raison, Abdullatif Aydin, Zhouyang Xu, Katie Zuo, Kawal Rhode, Antonia Pontiki

**Affiliations:** 1 Department of Surgical & Interventional Engineering, School of Biomedical Engineering and Imaging Sciences, King’s College London, London, UK; 2 MRC Centre for Transplantation, Guy’s Hospital, King’s College London, London, UK; 3 Department of Urology, Guy’s and St Thomas’ NHS Foundation Trust, London, UK; 4 King’s College London School of Medical Education, London, UK

**Keywords:** 3D model, Penile implant surgery, Simulator, Training, Evaluation

## Abstract

**Background:**

Penile implant surgery is the standard surgical treatment for end-stage erectile dysfunction. However, the growing complexity of modern high-tech penile prostheses has increased the demand for more practical training opportunities. The most advanced contemporary training methods involve simulation training using cadavers, with costs exceeding $5,000 per cadaver, inclusive of biohazard fees. This study introduces an innovative and cost-efficient male urogenital simulator designed to enhance penile implant surgery training.

**Methods:**

Utilizing image segmentation of patient pre-operative computed tomography (CT) scans, combined with three-dimensional (3D) printing and silicone molding techniques, we developed a high-fidelity simulator replicating the anatomical structures of the male urogenital system. The simulator incorporates an innovative double-layer structural design encompassing the corpus spongiosum and glans, corpora cavernosa, testes, epididymides, and pelvic bones. Additionally, it utilizes a two-stage skin manufacturing process tailored for different skin regions. The simulator was produced at a low material cost of £10, with an average production time of 3 h. To evaluate its training efficacy, we conducted a penile implant surgery training session involving 15 urology trainees and surgeons ranging from specialty training levels ST3 to ST6. The session began with a demonstration of penile implant surgery and error detection. Trainees, averaging three per simulator, practiced corporotomy, dilation, measurement, penile prosthesis, and scrotal pump placement under expert guidance. Participants’ feedback was collected using a Likert scale questionnaire, assessing learning, satisfaction, and anatomical accuracy.

**Results:**

Quantitative analysis of the questionnaire responses indicated highly positive feedback from the participants. Satisfaction rates surpassed 96% in learning effectiveness, over 89% in overall satisfaction, and 86% in anatomical accuracy demonstration. The simulator was favourably reviewed by both urology trainees and experienced surgeons, highlighting its utility as a practical training tool. Its low production cost and high precision make it a viable alternative to current training models.

**Conclusions:**

The development of this cost-efficient, anatomically accurate urogenital simulator through advanced imaging and additive manufacturing techniques represents a significant advancement in penile implant surgical training. This state-of-the-art simulator not only provides a realistic and practical training experience but also underscores the potential for 3D printing technologies to revolutionize medical education and training.

**Supplementary Information:**

The online version contains supplementary material available at 10.1186/s41205-024-00248-5.

## Background

 In recent years, the demand for effective surgical training tools has grown, driven by ethical considerations and technological advancements. Surgical simulators, particularly those utilizing additive manufacturing, offer a more ethical and cost-effective alternative to cadavers while providing high realism and fidelity. Appropriately designed simulators, when seamlessly incorporated into the educational framework, possess the capability to practice the surgical operation with realistic experiences that elicit or duplicate significant facets of real patient cases in a completely interactive fashion [1].

Urology has encountered a lack of simulation models tailored to its domain. Specifically, the simulation landscape in open urological surgery is particularly constrained, with a dearth of developed simulators [2]. Notably, in the realm of penile implant (PI) surgery aimed at rectifying erectile dysfunction, the intricacy inherent in procedures involving advanced penile prostheses emphasize the importance for surgeons to undergo comprehensive training. The conventional approach to teaching PI surgery has traditionally adhered to the ‘see one, do one, teach one’ model [2]. However, a paradigm shift is occurring as surgical simulators gain increasing popularity. This shift is driven by their ethical advantages and cost efficiency compared to the utilization of cadavers. In UK, the current simulation training typically involves the utilization of anthropomorphic dummies and virtual reality (VR) simulators. The anthropomorphic models currently employed in PI surgery primarily consist of a rigid plastic body. The unibody design of the penis and scrotum lacks internal anatomical structures, featuring only a urethra. The absence of functional representation hinders the effective simulation of the actual penile implant procedure. Conversely, VR simulators provide a 3D display of implant procedures but lack the realistic texture experienced during human tissue dissection. An anatomically accurate simulator with implant procedure practice functionality will enable surgeons to practice and improve their skills, as well as study a specific patient’s anatomy prior to performing a procedure. This anatomically accurate simulator can be achieved through additive manufacturing (AM), which has seen increased usage in healthcare, especially for the development of anatomical models. AM stands out for its cost-effectiveness, material versatility, wide availability, and quick production. Thus, it represents a suitable technology for the development of surgical simulators.

**Fig. 1:**
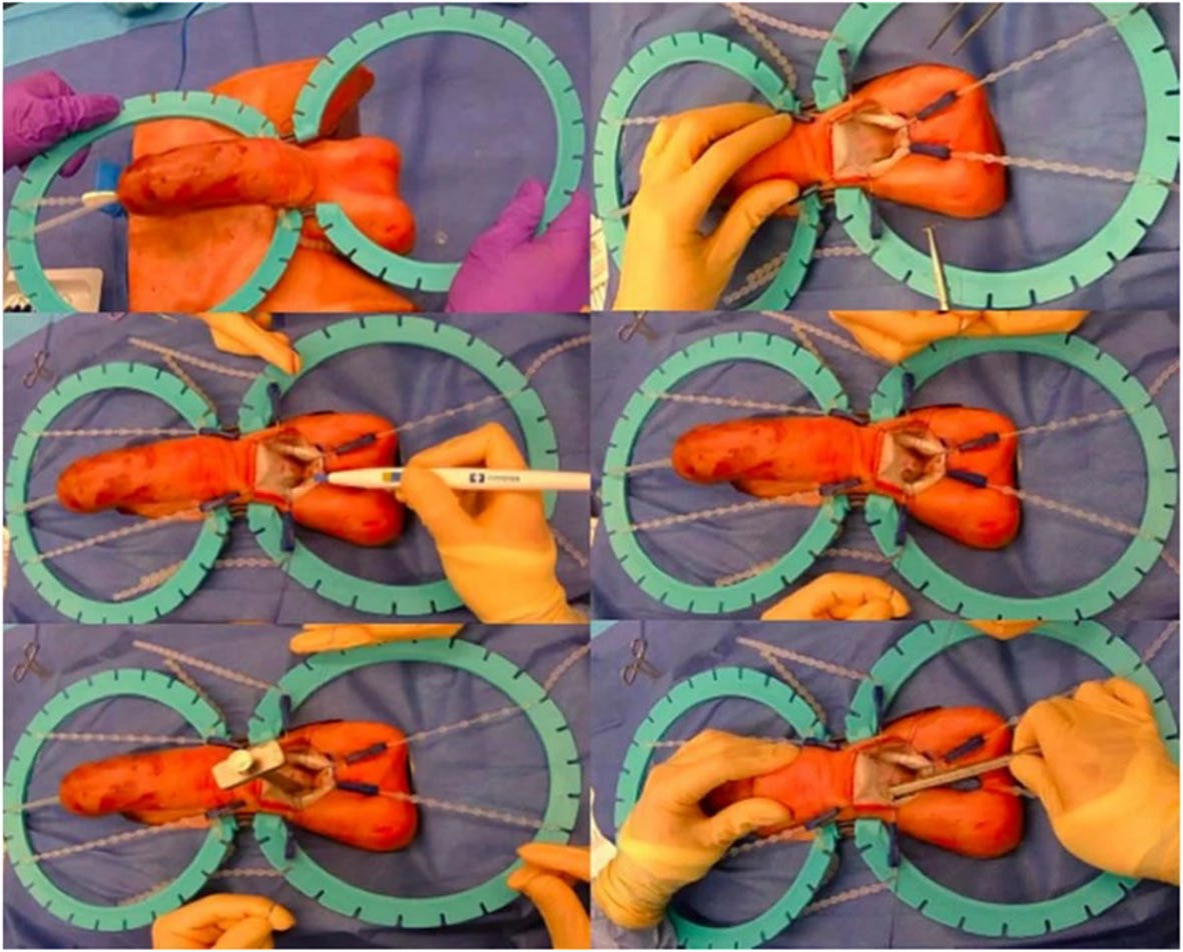
The 3D pelvic cadaver model developed by University of Rochester [4]

The requirements for a high-fidelity simulator for PI surgery are challenging. These requirements include representation of the male urogenital anatomy, suitability for a range of the steps of PI surgery (corporotomy, dilation, measurement, scrotal pump placement, reservoir placement, implantation), and error detection (corporal crossover, incorrect prostheses assembly). In 2020, a simulator was developed by University of Rochester that met most of the criteria [3]. Nevertheless, certain drawbacks remain apparent in this design. The replication of all soft anatomical structures within their simulator involved the use of polyvinyl alcohol (PVA) gel. This material has a maximum storage lifespan of one month after exposure to air. To extend the longevity of the PVA gel, it must be kept in a liquid state, resulting in a significant increase in both the storage and transportation expenses associated with the simulator. Our research aimed to develop a novel male urogenital simulator tailored for PI surgery training. The objective was to provide trainee urologists with a 3D physical model of the male urogenital anatomy, which is anatomically accurate, easily reproducible and has a high degree of fidelity and realism, while maintaining a low cost. Our study evaluated the simulator’s effectiveness, addressed key limitations of existing models, and it will contribute to the ongoing efforts to enhance surgical training in the evolving landscape of 3D printing and additive manufacturing (Fig. 1).

## Materials and methods

### Biomimetic material

Silicone rubbers and silicone foams are excellent biomimetic materials for creating a male urogenital simulator. These materials can be molded and shaped to closely resemble natural tissue’s texture, elasticity, and durability. Silicone foam can also be used to mimic the structure of tissues, such as the sponge-like tissue found in the corpus cavernosum of the penis. The Slacker solution was used as a softener to optimize the silicone material’s performance.

To ensure the simulator’s realism, potential materials were made into round samples with a one-centimeter thickness and assessed by a team of experienced urologists. The urologists assessed the material samples based on their tactile properties in comparison to actual human tissue, considering factors such as texture, elasticity, and hardness. The final choice of materials and mixing ratios was determined based on feedback from the urologists after evaluating each sample twice. The material selection, mixing ratios and cost are presented in Table 1.

**Table 1:** Composition of silicon materials (Smooth-on, PA, USA), for various anatomy structures in the simulator, including corresponding weights and material costs, with total material cost summarized at the table’s bottom row

Anatomy structure	Material Choice	Material Price per Kg (£)	Quantity	Cost per Anatomical Structure (£)
Corpus spongiosum & glans	Soma Foama 25	25.01	0.04 Kg	1.00
Tunica albuginea	DragonSkin FX pro	23.82	0.06 Kg	1.43
Corpus cavernosum inner	Modified Soma Foama 25	16.83	0.06 Kg	1.50
Neurovascular bundle	Colored wires	0.18/meter	0.60 m	0.11
Box fascia	EcoFlex Gel 2	20.90	0.04 Kg	0.84
Connective tissue	Silicon Adhesive	32.05/tube	1/20 tube	1.60
Penile skin	DragonSkin FX pro + slacker with ratio 2:1	24.61	0.06 Kg	1.43
Scrotal skin	DragonSkin FX pro + slacker with ratio 1.5:1	24.40	0.04 Kg	0.95
Testis	EcoFlex0030	19.54	0.03 Kg	0.58
Epididymis	EcoFlex0010	21.45	0.01 Kg	0.21
**Total**				**9.65**

### Imaging segmentation

**Fig. 2:**
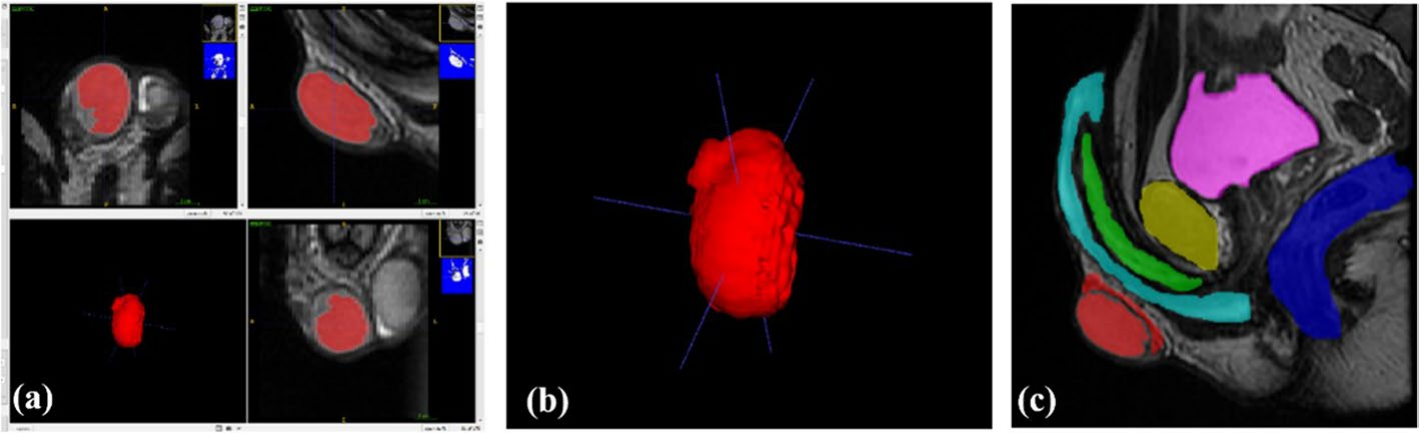
**a **Segmenting a single testis using a semi-automatic technique. **b** Initial segmentation of a single testis. **c** Segmentation of each desired anatomical structure. Red: Testis & Epididymis; Cyan: Corpus spongiosum and Glans; Green: Corpus cavernosum; Yellow: Prostate; Purple: Bladder; Blue: Rectum

Image segmentation of the male urogenital anatomical structures was performed on a series of Digital Imaging and Communication in Medicine (DICOM) formatted images obtained from an anonymized, pre-operative, male patient urogenital contrast MRI and CT scans. Both manual and semi-automatic segmentation features of ITK-SNAP (University of Pennsylvania, Philadelphia, PA, USA) were used to process the patient’s DICOM images. The software allowed for accurate segmentation of the male urogenital anatomy, which was then used to create a digital 3D model. The original segmentation of the testes and epididymides, corpus spongiosum and glans, corpora cavernosa and pelvic bones were obtained. Figure 2 illustrates the segmentation of the tissues.

**Fig. 3:**
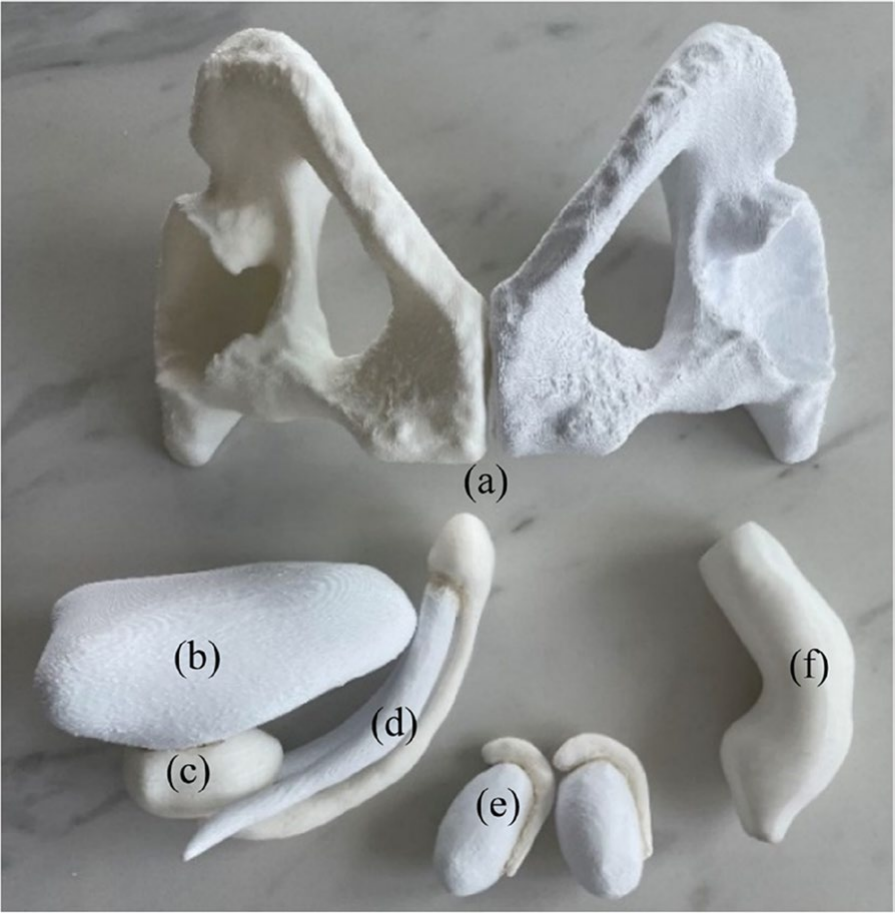
Individual 3D printed structures in PLA. (a) Pelvic bones (anterior view) (b) Bladder (c) Prostate (d) Penis (three Corporal bodies with Glans) (e) Testess with epididymides (f) Rectum

To ensure that the simulator would be adaptable to penile prostheses and have proper size and shape for simulation training, the 3D meshes of all the soft tissue structures were refined using the smoothing brush in Meshmixer (Autodesk, USA). Additionally, the shapes of these structures were adjusted while consulting with the urologists and the penile prosthesis developer industry partner to ensure the simulator was effective for training and education purposes. To better evaluate and improve the 3D digital anatomies, physical 3D printed prototypes of the segmented anatomical structures were created using polylactic acid (PLA) filament material (Fig. 3). The digital models were sliced using Ultimaker Cura 4.8.0 (Ultimaker, Utrecht, The Netherlands) and 3D printed by using a Chiron FDM 3D printer (Anycubic, Shenzhen, China). These physical models served as a visual representation of the digital model during the consultation process with the urology experts, which allowed them to evaluate and assess the accuracy of the model more directly.

**Fig. 4:**
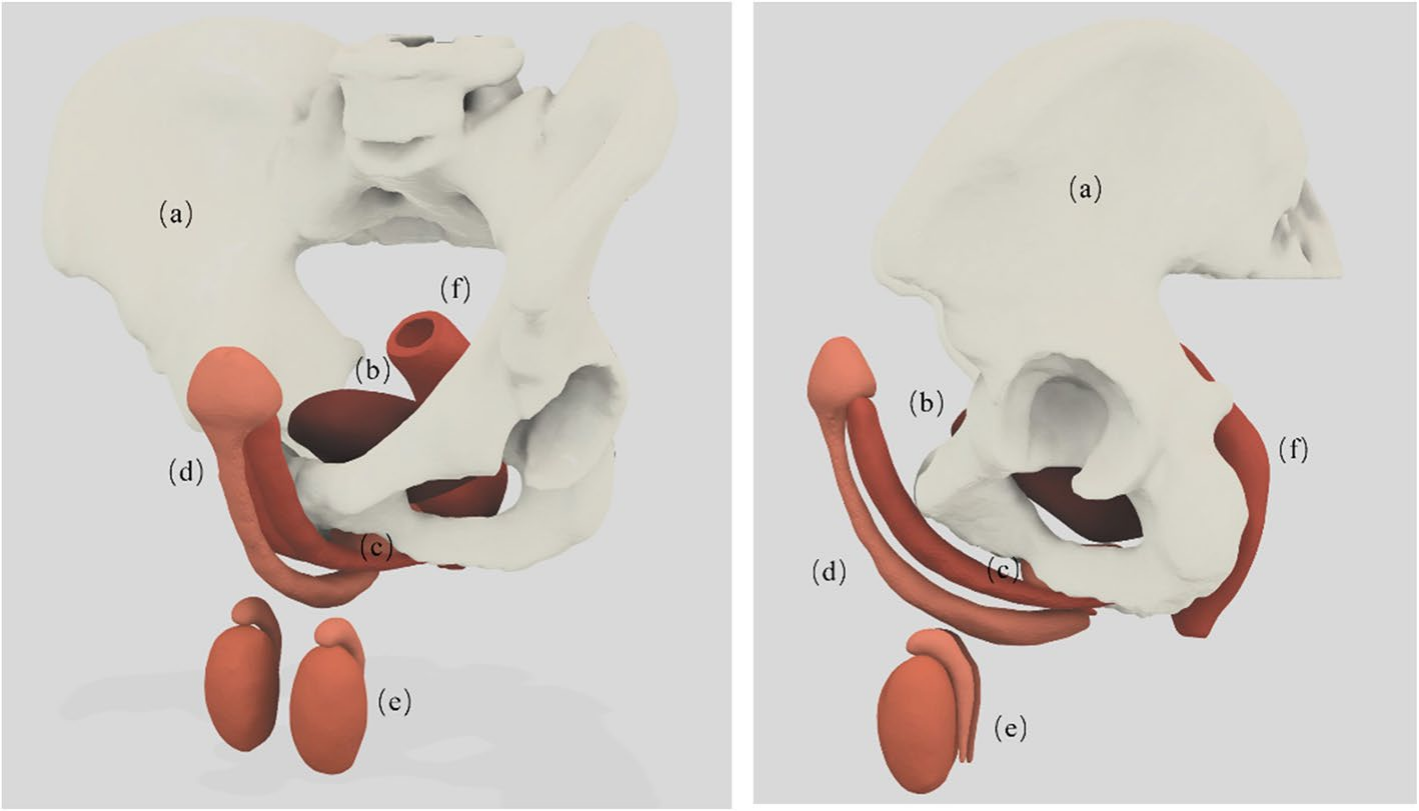
Computer model of the simulator with main anatomical structures. (a) Pelvic bones (b) Bladder (c) Prostate (d) Penis (three Corporal bodies with Glans) (e) Testes with epididymis (f) Rectum

The model was further adjusted in size and shape to ensure compatibility with penile prostheses. This was done by taking precise measurements of the prostheses provided by the industry partner and using this information to make the necessary adjustments to the corporal bodies. The goal was to accurately represent the average size and shape of the corporal bodies of a male. Figure 4 demonstrates all the anatomical structures that were processed using Meshmixer.

### Mold making

Once the size and shape of all anatomical structures were confirmed, each model was individually exported in STL format from Meshmixer. The exported model was then edited in Fusion360 (Autodesk, San Rafael, CA, USA). Utilizing Fusion360, a mold was created. This process was repeated for all anatomical structures, including the corpora cavernosa mold, corpus spongiosum & glans mold, a penis-scrotum external mold, and testis & epididymis mold. Furthermore, an innovative design was developed for the penis-scrotum external mold. The design included an additional hanging part that was carefully designed to recreate the hollow structure of the scrotum. Finally, the exported computer-designed molds were sliced using Cura and 3D printed in PLA using a Chiron FDM 3D printer.

### Simulator assembly

The manufacturing process for the penis and scrotum followed the principle of starting from the innermost structures to the outer structures. Based on the material choice (Table 1), the liquid silicone material was poured into the corresponding mold. During the curing process, the silicone material underwent a chemical reaction that caused it to cross-link and form a solid, durable material. The cure time of different materials varied; silicon foam took approximately 15 min, and silicon rubber took on average 1 to 1.5 h to cure.

The corpora cavernosa was manufactured using a novel sponge material. Concurrently, the tunica albuginea was created by applying a highly flexible and resilient silicon material coating.

The single corpus spongiosum and glans were manufactured with Soma Foama 25, which were connected with two corpora cavernosa using silicon adhesive, forming the basis of the penis body. The box fascia was made of Ecoflex Gel 2 that was specifically formulated and poured onto a flat surface. Additionally, on the side of the corpora cavernosa, three electric wires were glued onto the fascia layer to indicate the artery and vein in the penis region. Finally, all the parts, including the penis body basis, the fascia layer, and the electric wires, were placed in the penis-scrotum external mold. The mold represented the skin of the penis and scrotum. A two-stage skin manufacturing technology was utilized to recreate the different material properties of the penis and scrotum skin. This was achieved by first pouring DragonSkin FX into the mold to make the penis skin. Upon complete curing, DragonSkin FX, formulated with Slacker, was utilized to fill the remaining space within the mold, resulting in a connected penis and scrotum with different material properties.

For the internal structure of the scrotum, two testes and two epididymis were manufactured using the Ecoflex0010 and Ecoflex0030, which were then connected using silicon glue. A 3 mm diameter fishing wire was attached to the tail of the epididymis, indicating the spermatic cord. A similar fascia layer made of Ecoflex Gel 2 was then wrapped around the entire structure. The internal structure of the scrotum was designed to be placed in the scrotum room, created by the hanging part in the penis-scrotum external mold.

### Evaluation

To evaluate the efficacy of the male urogenital simulator, a quantitative trial was conducted at the Weston Education Centre, King’s College Hospital, involving 15 senior trainees, specialist urology trainees and surgeons spanning stages 3 to 6. Six simulators were specifically manufactured for this trial. The evaluation initiated with a skilled surgeon presenting a penile implant surgery demonstration, delivering a comprehensive lecture on the primary procedure, and elucidating error detection methodologies. Following the demonstration, trainees participated in personalized practice sessions, with an average of three trainees per simulator, under the guidance of experienced surgeons. These sessions involved practicing procedures such as corporotomy, dilation, measurement, penile prosthesis placement, and scrotal pump placement. Post the practical exercise, all participants completed a questionnaire encompassing 19 questions, evaluating dimensions of learning, overall satisfaction, and anatomical accuracy, rated on a Likert scale. Recorded data, treated as numerical variables, were stratified based on the quantity of surgeries conducted (0–250 and 250–1000) and the training stage (stage 3&4, and stage 5&6). The Mann-Whitney U test was utilized to examine noteworthy differences between the specified data groups. This non-parametric test was applied to Likert scale data, which did not follow a normal distribution. The test employed a two-tailed approach with a significance level (α) set at 0.05.

## Results

### Simulator demonstration

**Fig. 5:**
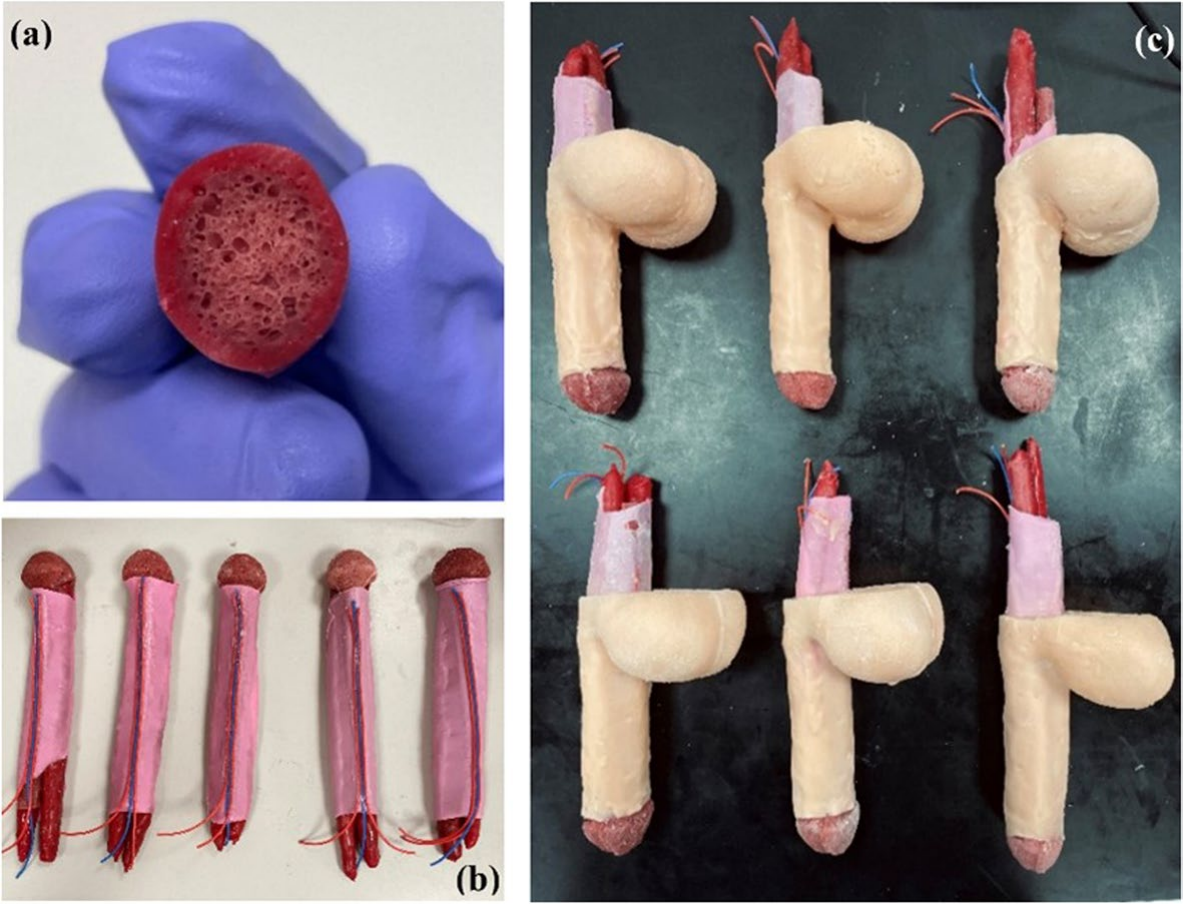
**a **Cross section of the corpus cavernosum and tunica albuginea featuring a novel double layer structure. **b** Assembled corporal bodies. **c** Assembled simulator

**Fig. 6:**
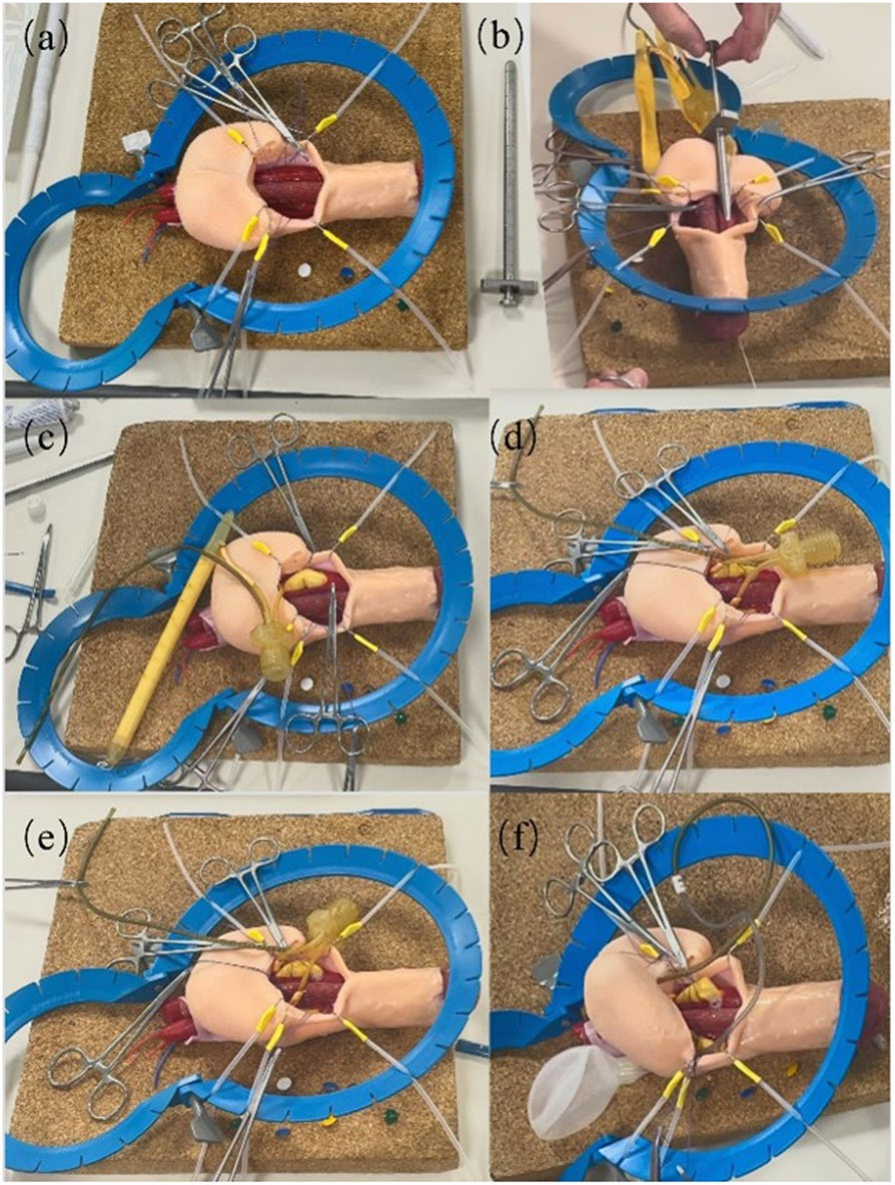
Simulator utilized in the urology training session at King’s College Hospital, demonstrating the progressive steps of the penile implant surgery procedure, including (**a**) corporotomy, (**b**) measurement, (**c**) & (**d**) penile cylinders placement, (**e**) & (**f**) scrotal pump placement

All primary organs and tissues constituting the penile and scrotal structures have been fabricated and integrated in accordance with precise anatomical configurations, aiming to yield a faithful representation. The production of the corpora cavernosa was a crucial step of the overall simulator construction, as it served as the primary site for penile prostheses placement. The most challenging aspect of implant surgery is the dilation of the erectile tissue inside the corpora cavernosa while maintaining the integrity of the outer layer. The successful assembly of the three corporal bodies, exemplified in Fig. 5, incorporates a dual-layer methodology utilizing an internal sponge to faithfully replicate the corpora cavernosa. The sponge material is denoted by the red region in Fig. 5a, while the external layer is sheathed in a highly elastic and durable silicone material. In order to emulate the fascia of the penis and testes, a thin silicon rubber sheet was employed, resulting in an exceptionally realistic representation (Fig. 5b). Figure 5c showcases the final simulator, distinguished by a two-stage skin manufacturing technology, imparting a smooth and authentic skin texture. This technological advancement facilitates the differentiation of material attributes between the penile and scrotal skin, endowing the former with heightened strength and the latter with increased softness, thereby enabling precise simulation of the tactile properties of penile skin. Additionally, a tricolored electrical wire was affixed to the fascial layer to accentuate the representation of arteries and veins within the penile region (Fig. 6).

### Evaluation feedback

**Fig. 7:**
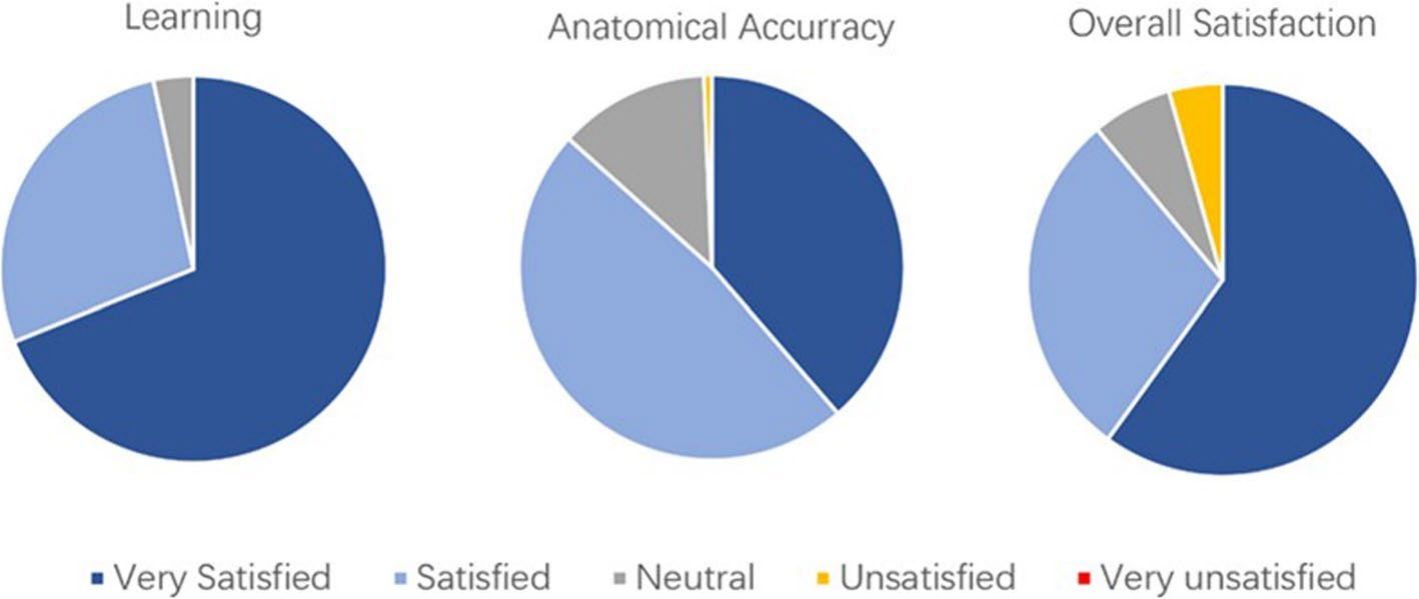
Pie Charts showing the qualitative results based on the feedback collected from the surgeons who completed the questionnaire. Left: Addressing the learning of the surgeons. Middle: The accuracy and quality of the model. Right: Overall satisfaction

Utilizing data derived from the feedback of respondents, Fig. 7 presents the percentage outcomes of satisfaction across three distinct categories. The outcomes are notably affirmative, with 96% of responses concurring that the simulator serves as a valuable and proficient training instrument for urology trainees, fostering enhanced confidence post-session. Respondents also acknowledge the simulator’s efficacy in facilitating male urogenital anatomy study and the practice of corporotomy, dilation, and inflatable penile implant replacement procedures. Remarkably, there is a complete satisfaction within the learning category. In terms of anatomical accuracy, 86% of responses indicate satisfaction, particularly concerning sensory and visual realism, with a singular dissatisfied response pertaining to the representation of the penis and scrotum skin. Within the overall satisfaction category, 89% of feedback expresses contentment with the simulator holistically. Furthermore, all trainees and surgeons express a desire to incorporate this simulator into their training programs.

Feedback was also categorized based on the trainees’ respective training stages, differentiating between stage 3 & 4 and stage 5 & 6. Following the performance of the Mann-Whitney U test, results indicated that, across all three categories, the outcomes from the higher-stage group (Stage 5 & Stage 6) did not significantly differ from those of the lower-stage group (Stage 3 & Stage 4). Conversely, when feedback was stratified based on the quantity of surgeries conducted, notable distinctions emerged. In the category of anatomical accuracy, the higher-quantity group (250–1000) exhibited superior results compared to the lower-quantity group (0–250). However, in the categories of learning and overall satisfaction, no statistically significant differences were observed. This implies that more experienced surgeons provide more positive feedback, underscoring the success in achieving the goal of enhanced anatomical accuracy.

## Discussion

The current penile simulator offers a high level of anatomical accuracy and practicality for corporotomy, dilation procedures and penile prosthesis placement in penile implant surgery. Compared to the simulator developed by the University of Rochester, our simulator offers several advantages. Firstly, the use of silicone material in our simulator offers several benefits over PVA gel. Silicone is more durable, flexible, and can maintain its shape and properties over time, making it a more reliable biomimetic material. The second advantage of our simulator is its reliance on the patient’s DICOM image, which results in a more precise reconstruction of male urogenital anatomy compared to the current simulator [3]. Third, the double-layer structure design of the corpora cavernosa and tunica albuginea in our simulator accurately replicates the structure and strength of the real cavernosa, enabling it to perform dilation procedures while maintaining correct penile anatomy. Furthermore, our simulator’s two-stage skin manufacturing technology results in a more realistic replication of human skin by allowing the penis and scrotum to have different material properties while being connected. Lastly, our simulator utilizes cost-effective materials significantly reducing manufacturing costs without compromising effectiveness. The materials cost of a single simulator is approximately £10. In comparison, the price of the Rochester model is €2000, which is considered high given that it is a one-time use model exclusively designed for PI surgery. Additionally, the standard cost of cadaveric simulation training in urology is lower, as expenses can be reduced by using unused cadaver body parts for other surgical procedures and utilizing a single cadaver for multiple residents. For example, in 2017, the Society of Urologic Prosthetic Surgeons and the Sexual Medicine Society of North America conducted cadaveric laboratory training for PI surgery. The total cost of this simulation training course was approximately $1,483 per resident [4]. Additionally, in the UK, a cadaveric simulation training course offered by the King’s Health Partners incurs a fee of £750 per resident [5, 6]. Overall, the simulator we developed is state-of-the-art and has the potential to significantly improve PI surgical training. One senior manager of the urology medical education team in Boston Scientific commented, “The work being undertaken with this study, it is envisaged, will assist in closing this identified gap and therefore hopefully shorten learning curves, improve surgical technique and lead to better patient outcomes.” However, based on the feedback, the current simulator model has limitations that need to be addressed. The primary limitation of the current model lies in the underdeveloped torso part of the simulator, which is crucial for simulating the reservoir placement of inflatable penile implant surgery. Additionally, as it is currently designed, the simulator is not reusable, which adds to its limitations. However, the platinum silicone material used in this research is eco-friendly and can be recycled through local recycling programs. On the other hand, the missing torso part, which is currently under development, will be a long-lasting component. The design will allow the penis part to be replaced for each training session, while the torso part remains reusable. Moreover, the absence of a urethral structure renders the simulator inadequate for practicing urethral catheter insertion. Subsequent efforts will concentrate on addressing these limitations and subjecting the enhanced simulator to a comprehensive surgical evaluation trial featuring comparative assessments.

In the future, the manufacturing methodology employed for this simulator holds the potential not only for application in penile implant surgery but also for the creation of models for surgical planning of complex cases such as kidney cancer surgery. Additionally, VR simulators might be hindered due to the lack of sufficient force data, which is challenging to collect during real patient procedures. This simulator, on the other hand, can recreate the tactile properties of tissues and the hands-on feeling of implant procedures. This feature positions it as a valuable tool not only for penile implant surgery but also for addressing the limitations faced by VR simulators in collecting force data during various urological surgeries, thereby offering an innovative solution for comprehensive surgical education and training.

## Conclusions

The present male urogenital simulator,, constitutes a highly precise and practical instrument for corporotomy, dilation, and penile prosthesis placement procedures in penile implant surgery. The amalgamation of anatomical accuracy and 3D printing technology played a crucial role in the simulator’s realization. The innovative double-layer corpora cavernosa, and adoption of two-stage skin manufacturing technology collectively contribute to improving the practice experience. The learning ability and anatomical accuracy are recognized favorably by urology trainees and surgeons; the simulator boasts a notably low materials cost compared to contemporary models while effectively fulfilling diverse functions. Overall, this state-of-the-art simulator has the potential to enhance penile implant surgical training and advance the application of 3D printing in medicine.

## Supplementary Information

Supplementary Material 1.

## Data Availability

No datasets were generated or analysed during the current study.

## Abbreviations

CT: Computed Tomography
MRI: Magnetic Resonance Imaging
3D: Three-dimensional
ST: Specialty Training
PI: Penile Implant
VR: Virtual Reality
AM: Additive Manufacturing
PVA: Polyvinyl Alcohol
BAUS: British Association of Urological Surgeons
DICOM: Digital Imaging and Communication in Medicine
PLA: Polylactic Acid
FDM: Fused Deposition Modeling
