# Supplementary material for: Low-cost male urogenital simulator for penile implant surgery training: a 3D printing approach
Source: 3D Print Med. 2025 Jan 3;11:1. doi: 10.1186/s41205-024-00248-5 (PMC11726939; doi:10.1186/s41205-024-00248-5)

Here are the uncropped images used in **Figure 6**. All other images or diagrams used in the manuscript have not been cropped.


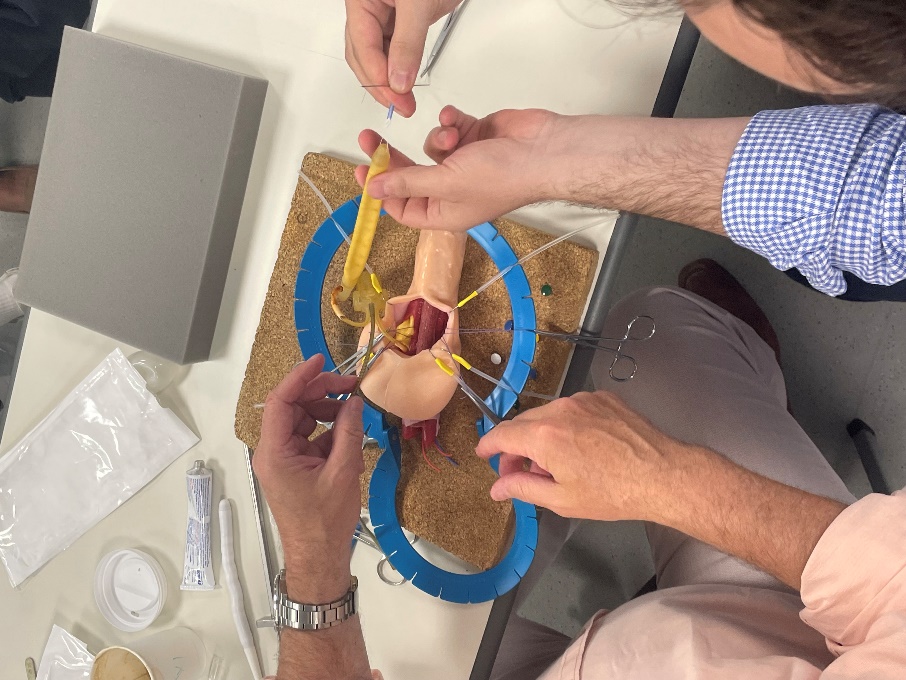


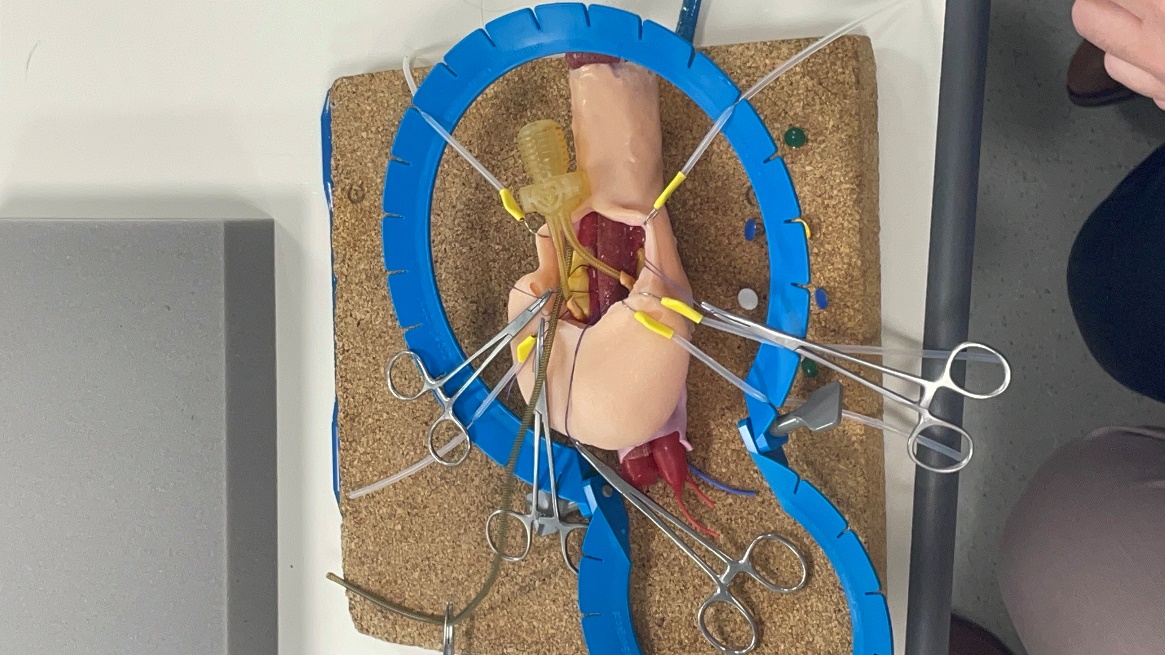


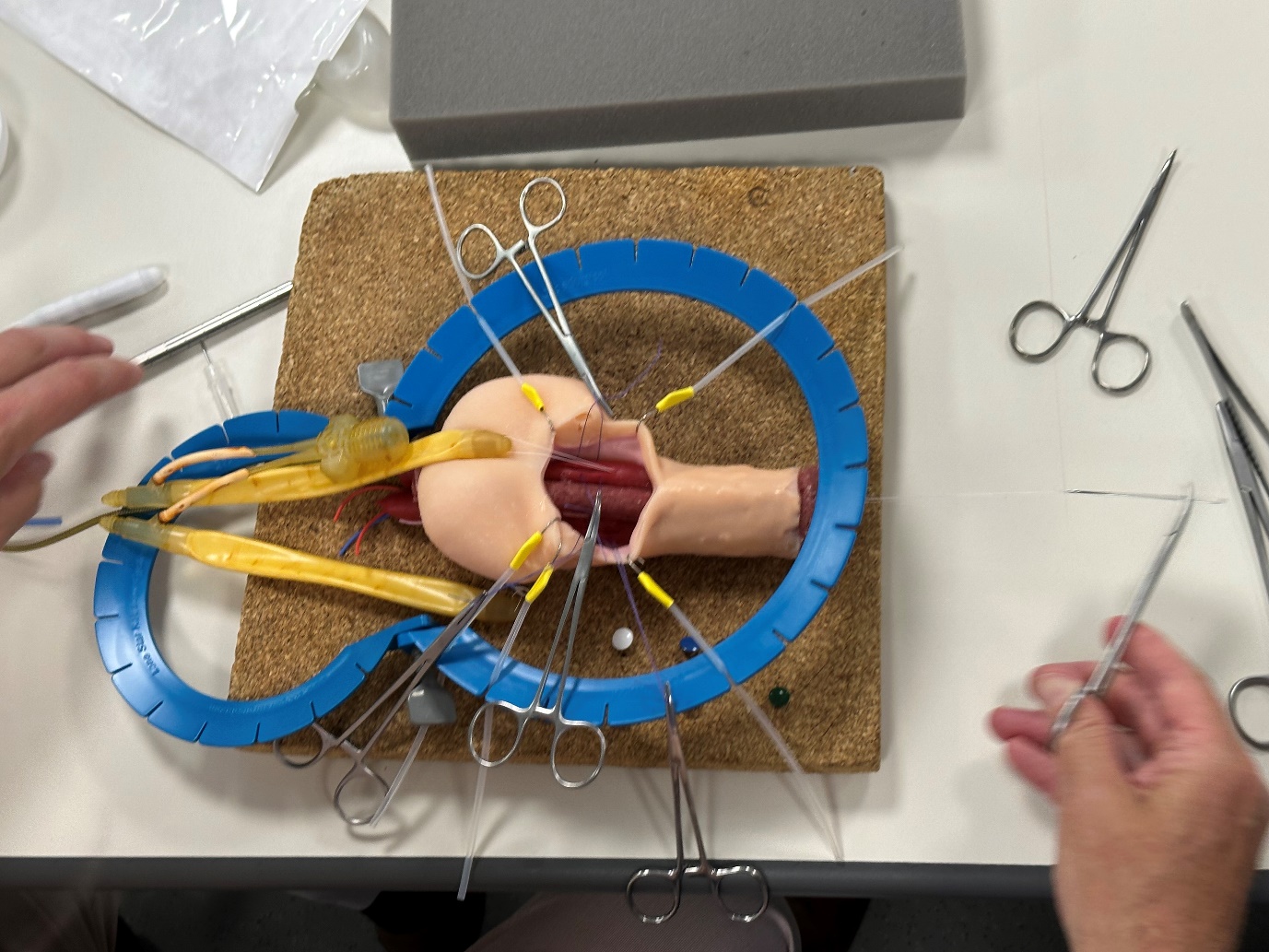


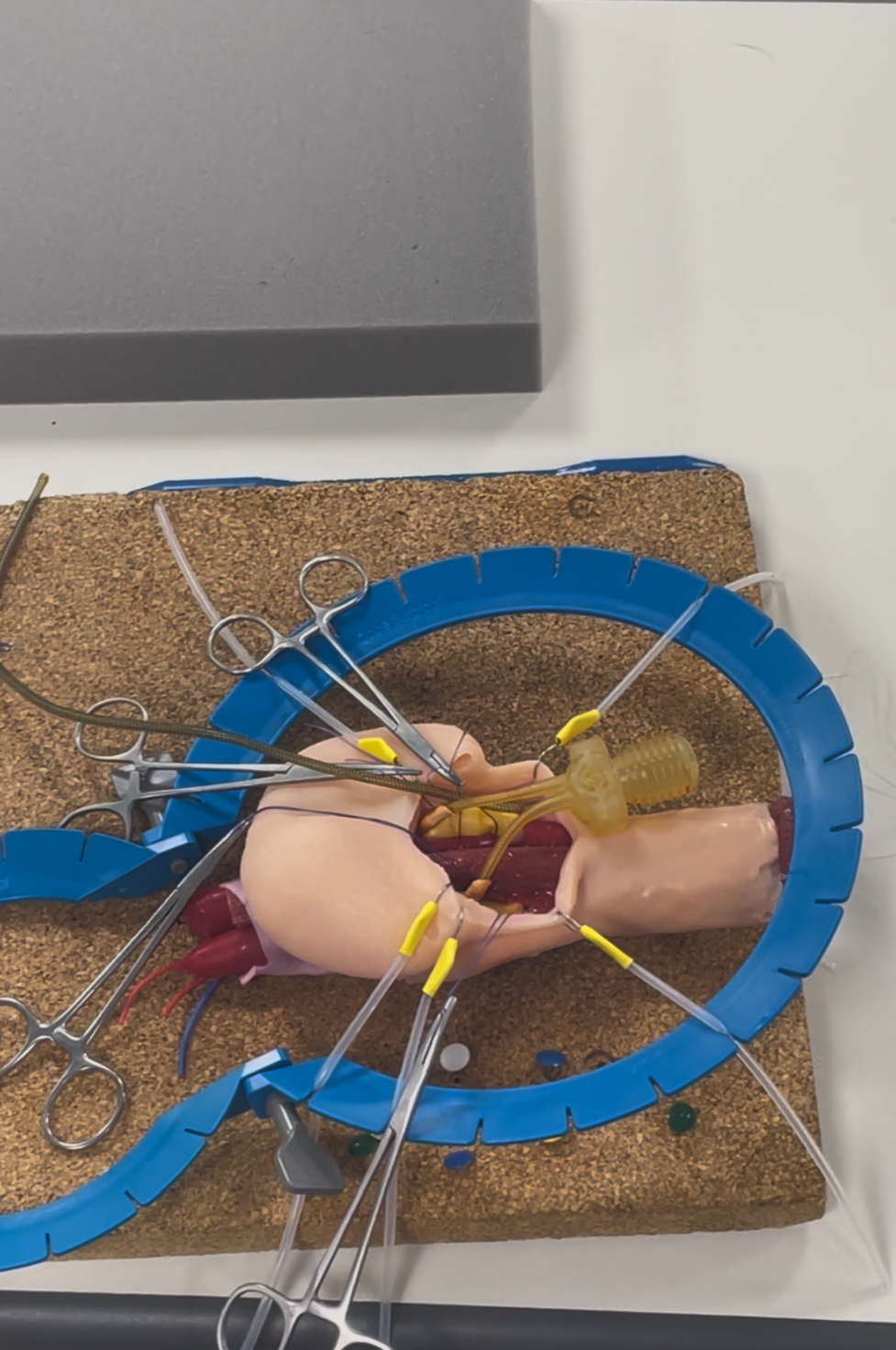


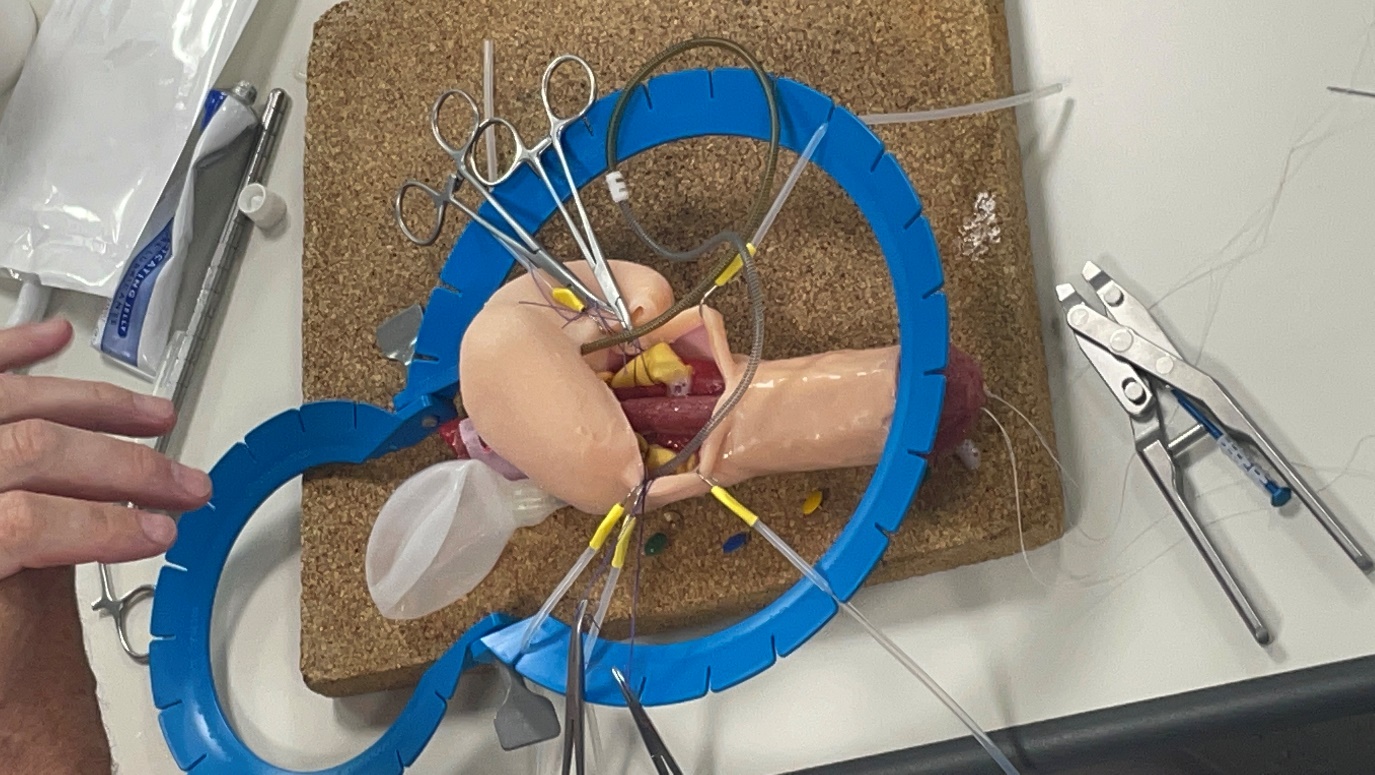

Supplement: Supplementary file 1 — Supplementary Material 1. [file 41205_2024_248_MOESM1_ESM.docx]
